# Neural Development – one year on

**DOI:** 10.1186/1749-8104-3-1

**Published:** 2008-01-09

**Authors:** Andrew Lumsden, Bill Harris, Joshua R Sanes, Rachel Wong

**Affiliations:** 1 MRC Centre for Developmental Neurobiology, King's College London, Guy's Hospital, London SE1 1UL, UK; 2 Department of Physiology, Development and Neuroscience, Cambridge University, Downing St, Cambridge CB2 3DY, UK; 3 Department of Molecular and Cellular Biology and Center for Brain Science, Harvard University, 7 Divinity Ave, Cambridge MA 02138, USA; 4 Department of Biological Structure, University of Washington, HSB G514, Seattle, Washington 98195-7420, USA

*Neural Development *was launched a year ago by BioMed Central. The Editors-in-Chief and the Publishing Editor for BioMed Central, Nandita Quaderi, hoped to fill a vacant niche in journal coverage and thereby provide a vehicle for outstanding work in the field. At the same time we wanted to take full advantage of the web-based, open-access publishing expertise of BioMed Central. As we celebrate our first birthday, we take this opportunity to reflect on our progress to date.

*Neural Development *is off to an excellent start, with an increasing number of papers being published each month and a quick turnaround. It is gratifying to see that work published in *Neural Development *covers the breadth of the field and comes from labs around the world. Articles range from molecular and cellular studies of neurogenesis and neuronal polarization from the groups of Chris Doe [1], Gordon Fishell [2] and Carol Schuurmans [3], through studies on axonal pathfinding by olfactory neurons by Liqun Luo [4] and motor neurons by Ed Laufer [5], to network functional studies from Jean Champagnat [6] and Alain Ghysen [7]. By virtue of their recent publication, articles have yet to be highly cited, but are actively downloaded. For example, an article from Bill Harris' lab [8] on polarization and orientation of retinal ganglion cells has been downloaded more than 6,000 times from the *ND *site alone. This paper also won the BioMed Central Research Award for Biology in 2007. Also highly accessed are papers from the groups of Angela Giangrande [9], Andreas Prokop [10] and Ahmed Mansouri [11].

That there was a need for this journal is shown by its becoming firmly established as a must-read within the community. For example, we heard much enthusiasm and strong support for the journal at this year's meeting of the Society for Neuroscience. As one of our colleagues put it, "this is *the *journal for the field." It provides the burgeoning population of developmental neurobiologists with exactly what they have wanted for some time – a journal dedicated to their branch of neuroscience, with an editorial board of experts from across the full breadth of the field, and rapid publication. In addition, open access means particularly high visibility for publications, as the full text is instantly available to anyone with access to a computer. Indeed, open access via the internet has enabled unprecedented availability for scientific work, with the widest possible distribution and readership of an article. This is in the best interests of not only the reader and the author, but also the latter's institute and the funding body. Playing to the strengths of on-line publication, we can also embed clickable movies within an article, a feature already used by a number of our authors. Students and postdocs will also find it useful that our on-line software makes it possible for every article to be a "cover article".

A very recent and exciting development is that *Neural Development *has now joined the Neuroscience Peer Review Consortium (NPRC). This new, experimental programme is intended to shortcut the all-too-frequent and all-too-painful process in which perfectly good papers are viewed as inappropriate for one journal, then need to be resubmitted and re-reviewed elsewhere, often with only minor modifications. The NPRC allows a paper found unsuitable for one journal to be passed, together with its referee reports, to another journal in the consortium. Of course it is entirely up to authors to decide whether to use NPRC and, if they do choose it, to decide where the paper should be redirected. Referees would also need to consent to having their comments passed on, and editors would still be free to select other referees, as considered appropriate. We expect this system will make life easier for authors, who may get their papers published with fewer rounds of review, and for referees, who would be relieved of having to review the same paper repeatedly. NPRC consortium members for the one-year trial period currently include *Journal of Neuroscience*, *European Journal of Neuroscience*, *Journal of Comparative Neurology*, *Neuroscience*, *Journal of Neurophysiology*, and *Neuroendocrinology*. It seems likely that at least a few others will join soon.

There are a few areas in which we see a clear need to improve. First, while we receive a healthy flow of papers on a range of early developmental topics, we get rather fewer than hoped for on later aspects of development – synapse formation, circuit formation and remodelling, and ontogenesis of behavior. We hope that a balance across the field will soon be achieved. Second, we hope that the freedom of an on-line format encourages two other forms of communication – reviews and meeting reports. A form of review we see as being particularly useful to the field is a head-to-head from key proponents of contemporarily contentious issues. Meeting reports (see, for example, [12]) are also valuable, as they bring research developments more rapidly to our audience than do either papers or reviews. Finally, we want to decrease the time from submission to first decision – at present this averages 4 weeks, which is competitive with other on-line journals but will be reduced further by ongoing streamlining in the editorial offices. As mentioned above, membership in the NPRC should also be a big help in this regard.

For many authors, readers and institutions there is one measure that matters above all others in assessing the status of a journal: the impact factor, as determined by The Institute for Scientific Information (ISI). The limitations of this metric are legion [13-15] but we know it is used as a criterion by hiring, funding and promotion committees – more so in some countries than in others. With this in mind, it is relevant to mention that BioMed Central is working closely with the Institute for Scientific Information [16] to ensure that citation analysis of articles published in *Neural Development *will be available. Although their ratings will not be available for some time, we expect they will be high, based on other indicators – for example, the high proportion of *Neural Development *papers selected by F1000 [17]. Moreover, by adopting an open access approach and making all articles freely available online we ensure that they achieve maximum visibility and impact. Our experience is consistent with recent findings demonstrating that open access articles are cited more quickly and more frequently than non-open access articles published in the same journal [18].

In short, we have had a good year and look forward to an even better one. We would be delighted to hear any suggestions you may have on how to improve the service. We thank you for your contributions – whether by submissions or refereeing, you are helping to create an invaluable resource for the scientific community.
